# Supplementary figures and images for: Modelling conformational state dynamics and its role on infection for SARS-CoV-2 Spike protein variants
Source: PLoS Comput Biol. 2021 Aug 5;17(8):e1009286. doi: 10.1371/journal.pcbi.1009286 (PMC8384204; doi:10.1371/journal.pcbi.1009286)

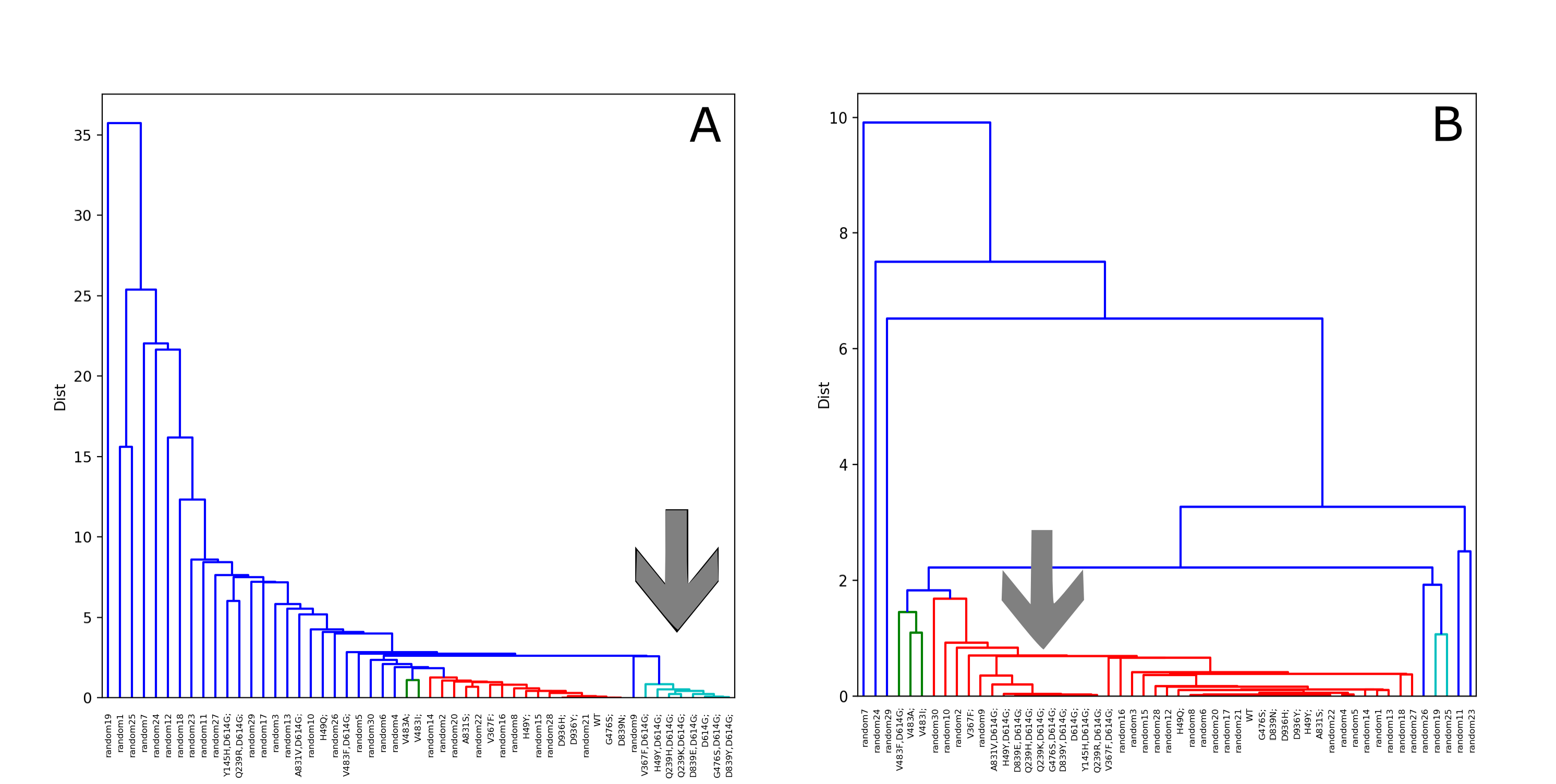

Supplement: S1 Fig — Dynamical Signature clustering between WT, 22 mutants observed in nature and 30 random mutants designed, both for the full structure (A) and only for the RBD (B). The grey arrow highlights the clusters containing most of the mutants that have the mutation D614G. (TIF) [file pcbi.1009286.s001.tif]

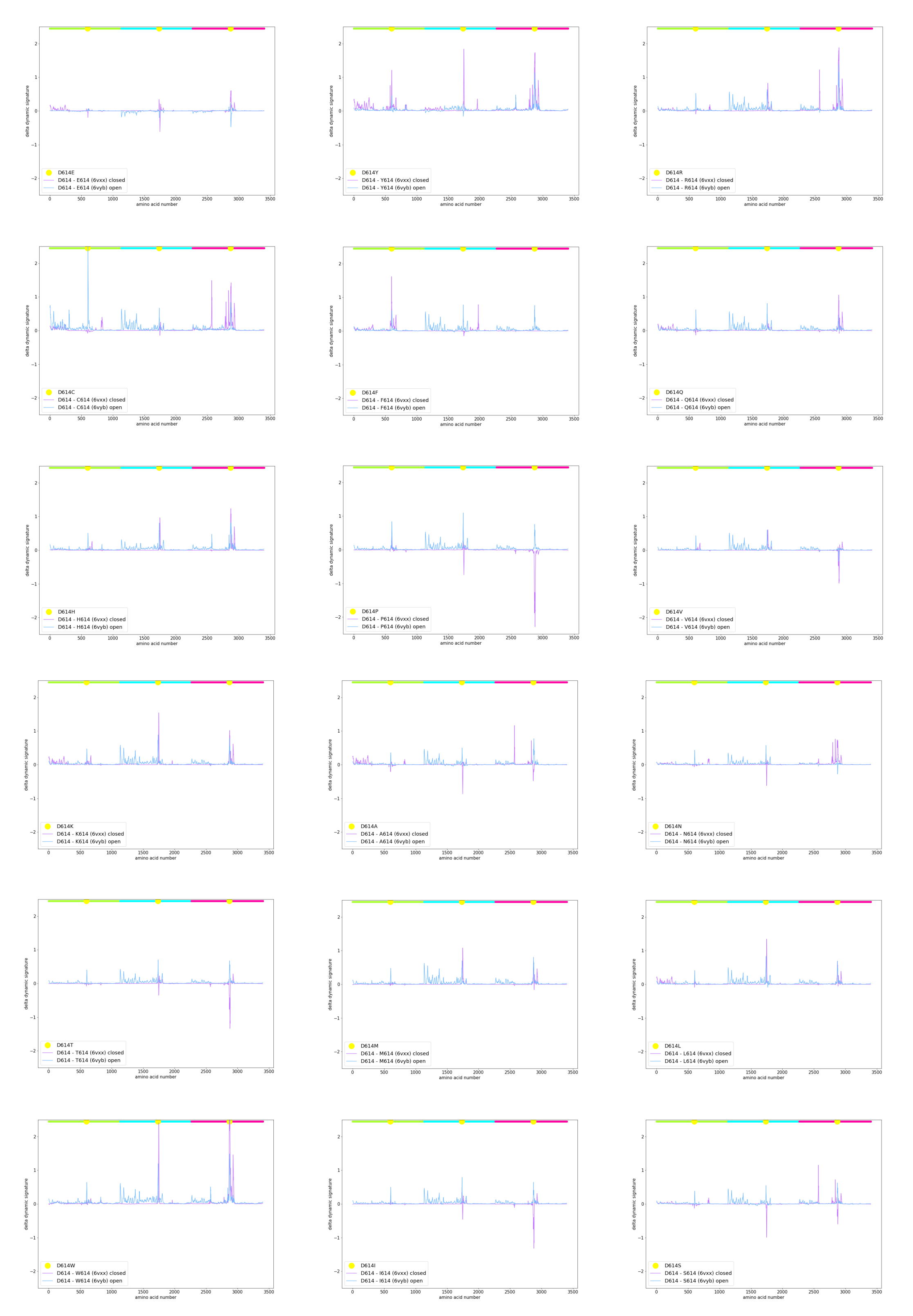

Supplement: S2 Fig — (TIF) [file pcbi.1009286.s002.tif]

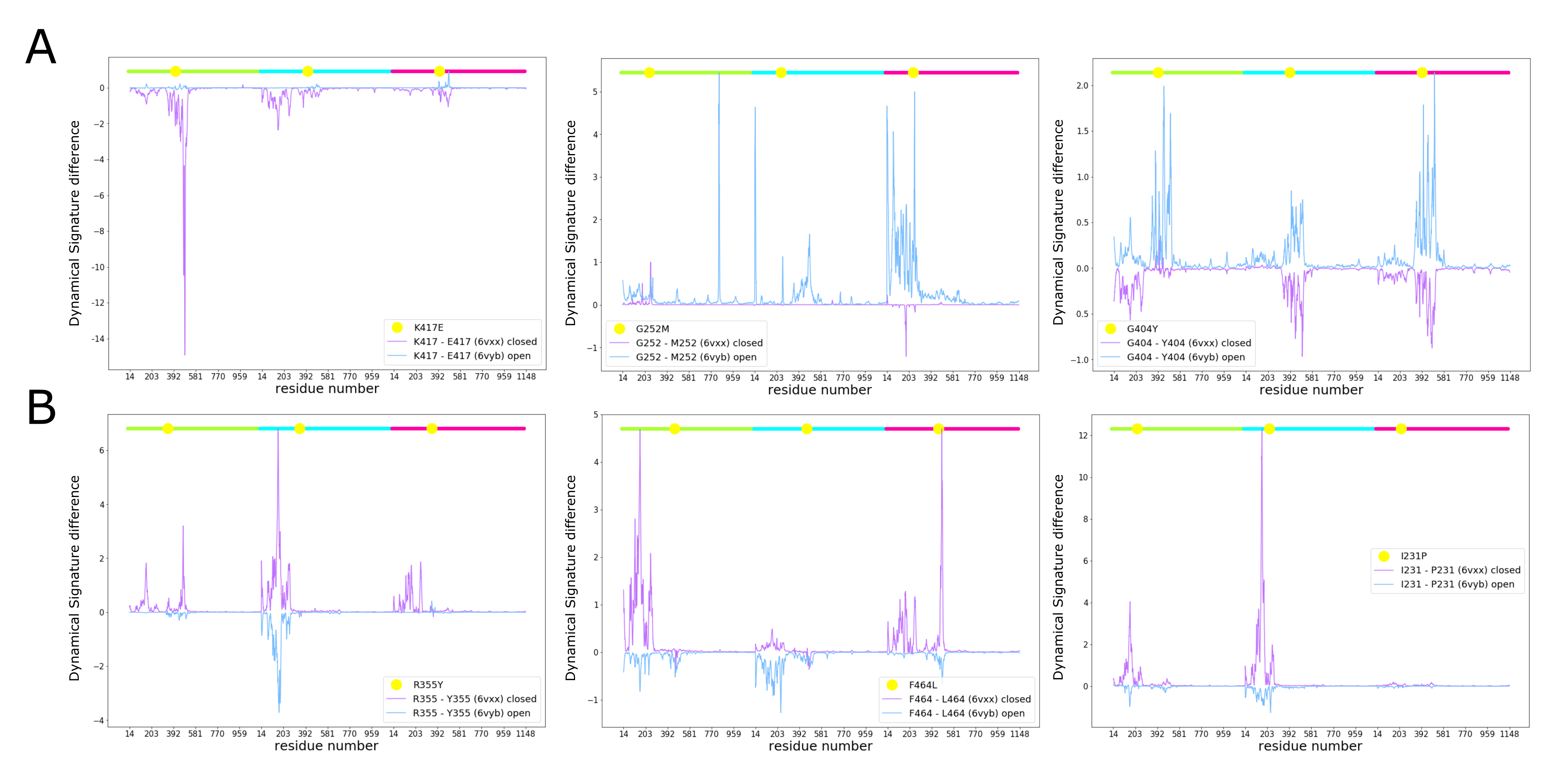

Supplement: S3 Fig — Dynamical Signature differences for three mutations among the top VDS (A)–K417E, G252M and G404Y –and bottom VDS values (B)–R355Y, F464L and I231P –for closed (purple) and open (blue) B chain RBD structures. Chains A, B and C are marked on the top in green, cyan and magenta, respectively, and the point mutations are marked in yellow. (TIF) [file pcbi.1009286.s003.tif]

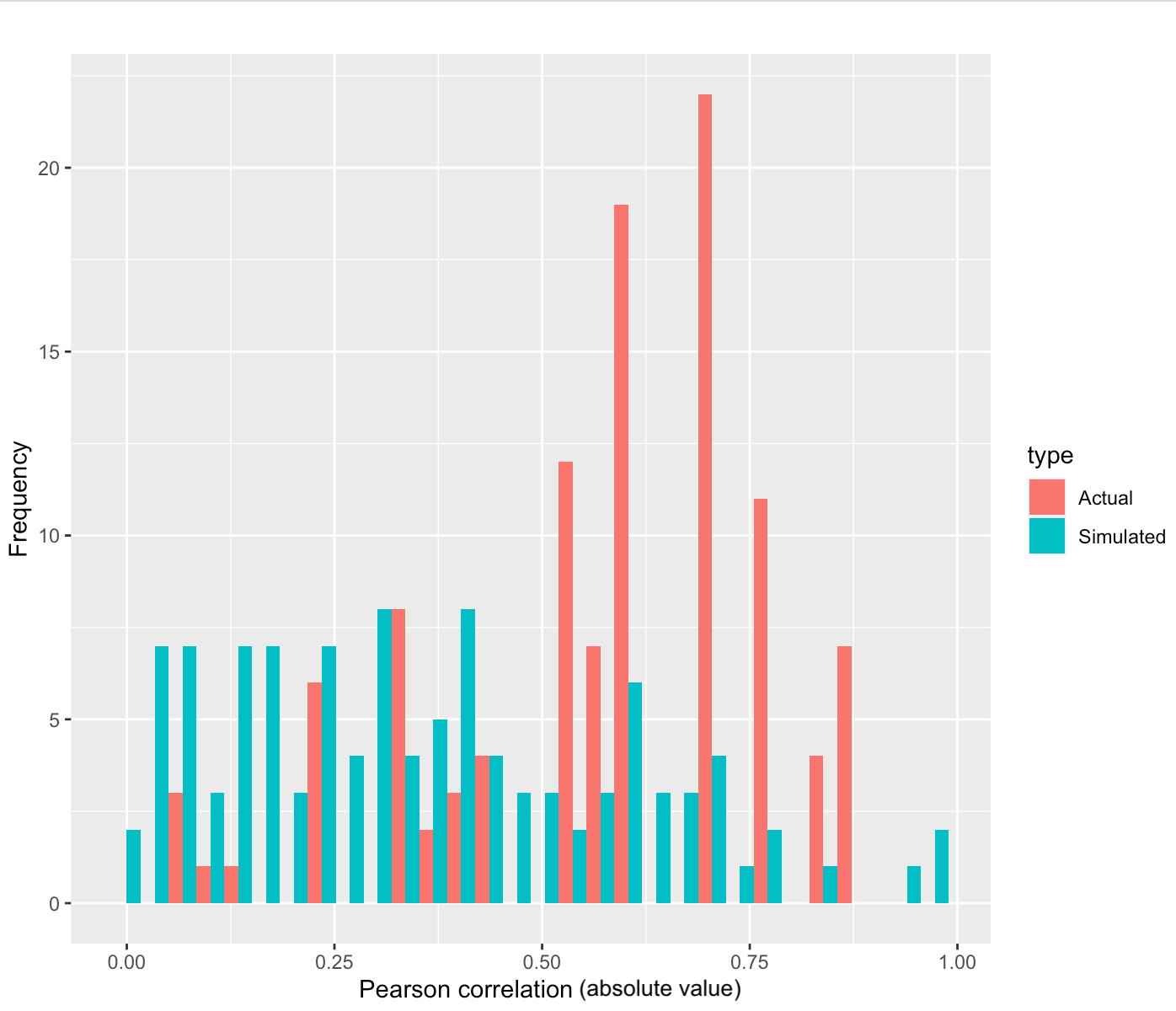

Supplement: S4 Fig — (TIF) [file pcbi.1009286.s004.tif]

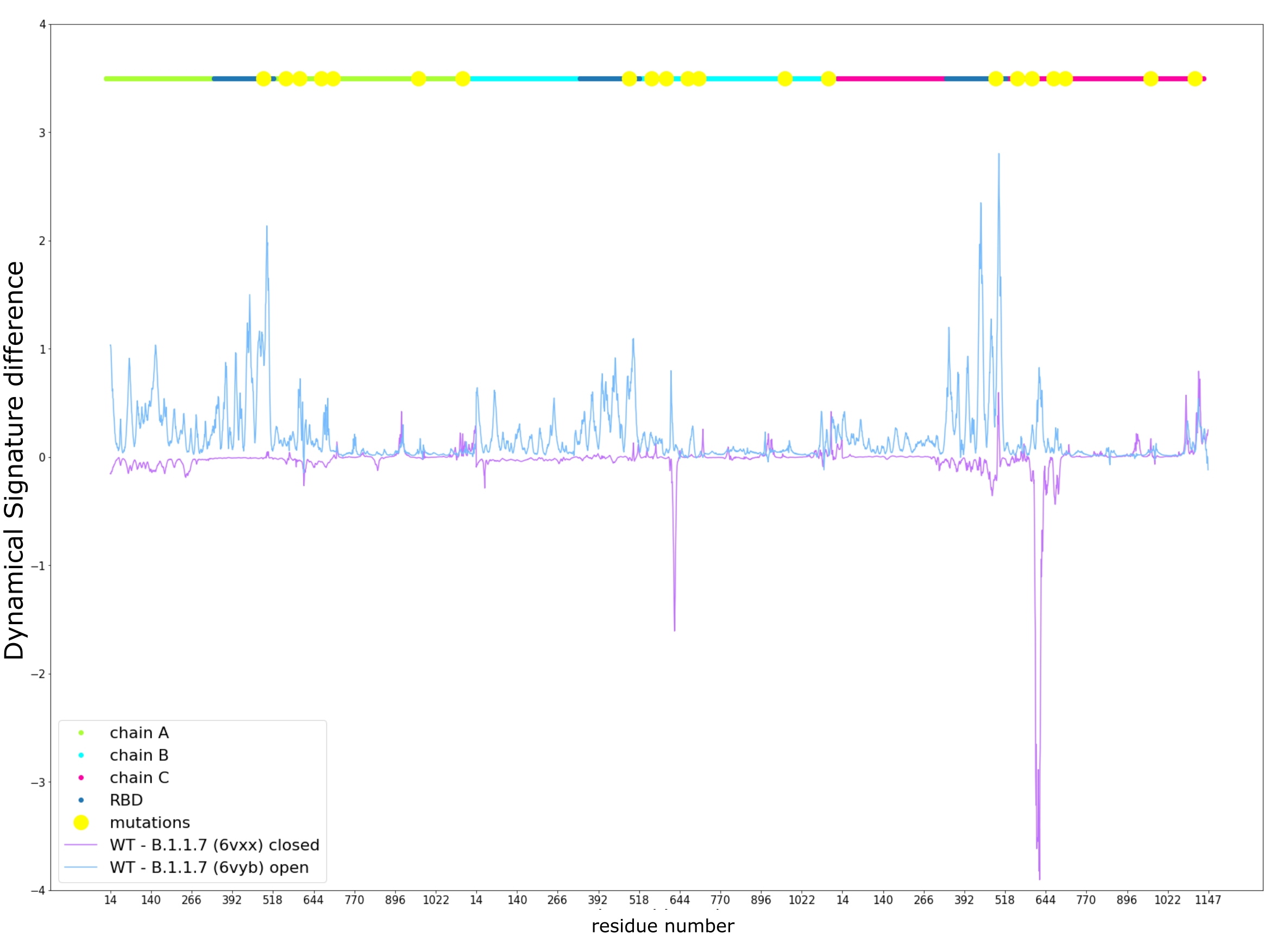

Supplement: S5 Fig — (TIF) [file pcbi.1009286.s005.tif]

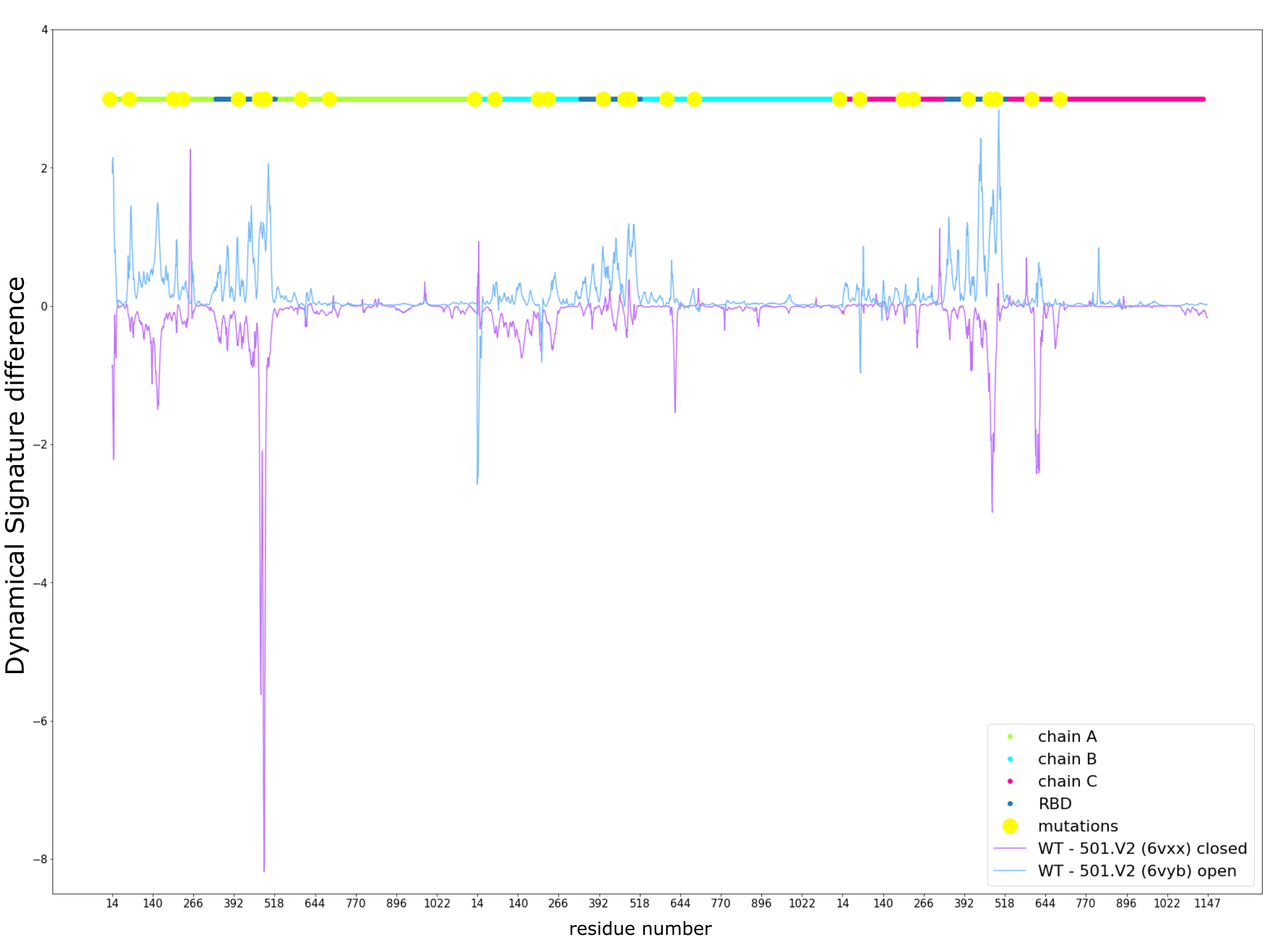

Supplement: S6 Fig — (TIF) [file pcbi.1009286.s006.tif]

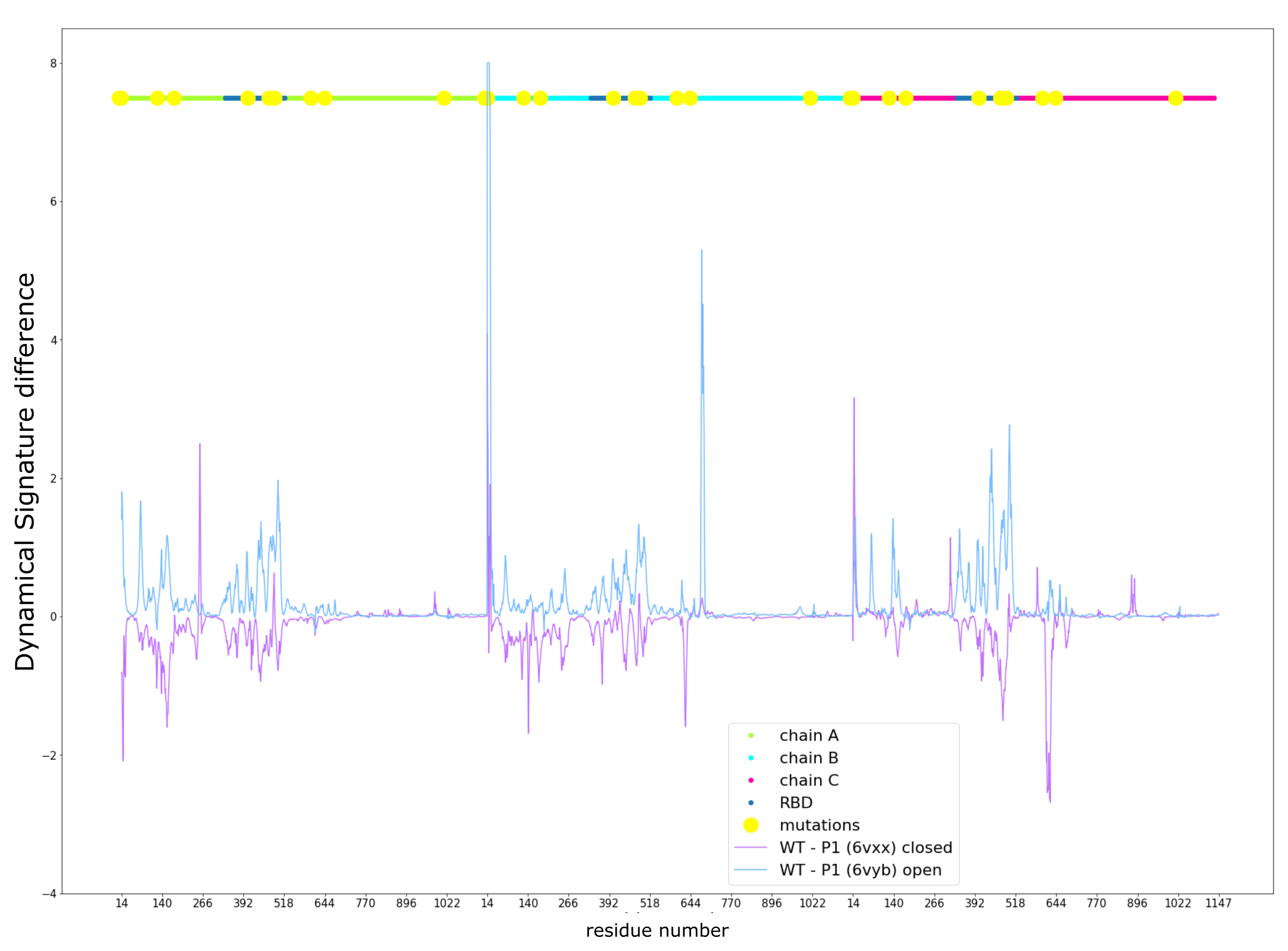

Supplement: S7 Fig — (TIF) [file pcbi.1009286.s007.tif]
