# Supplementary material for: Modelling conformational state dynamics and its role on infection for SARS-CoV-2 Spike protein variants
Source: PLoS Comput Biol. 2021 Aug 5;17(8):e1009286. doi: 10.1371/journal.pcbi.1009286 (PMC8384204; doi:10.1371/journal.pcbi.1009286)
Supplement: S1 Table — (DOCX) [file pcbi.1009286.s008.docx]

| L5F | Mutation not performed – out of range |
| --- | --- |
| L5F, G476S | Mutation not performed – out of range |
| L5F, D614G | Mutation not performed – out of range |
| L5F, D614G, D839Y | Mutation not performed – out of range |
| L5X | Mutation not performed – out of range |
| L5X, D614G | Mutation not performed – out of range |
| L8V | Mutation not performed – out of range |
| L8V, P1263L | Mutation not performed – out of range |
| L8W, D614G | Mutation not performed – out of range |
| L8X, D614G | Mutation not performed – out of range |
| H49Q | Mutation performed |
| H49X, D614X | Mutation not performed – nonsense mutation |
| H49X, D614G | Mutation not performed – nonsense mutation |
| H49Y | Mutation performed |
| H49Y, D614G | Mutation performed |
| Y145H, D614G | Mutation performed |
| Q239H, D614G | Mutation performed |
| Q239K, D614G | Mutation performed |
| Q239R, D614G | Mutation performed |
| Q239X, D614G | Mutation not performed – nonsense mutation |
| V367F | Mutation performed |
| V367X | Mutation not performed – nonsense mutation |
| V367F, D614G | Mutation performed |
| G476S | Mutation performed |
| G476S, D614G | Mutation performed |
| V483A | Mutation performed |
| V483F, D614G | Mutation performed |
| V483I | Mutation performed |
| V483X, D614G | Mutation not performed – nonsense mutation |
| D614G | Mutation performed |
| A831S | Mutation performed |
| D614G, A831V | Mutation performed |
| D614G, D839E | Mutation performed |
| D839N | Mutation performed |
| D839X | Mutation not performed – nonsense mutation |
| D614X, D839X | Mutation not performed – nonsense mutation |
| D614G, D839Y | Mutation performed |
| D936H | Mutation performed |
| D936X | Mutation not performed – nonsense mutation |
| D936Y | Mutation performed |
| D614G, P1263L | Mutation not performed – out of range |
| P1263L | Mutation not performed – out of range |
| D614G, P1263X | Mutation not performed – out of range |

**S1 Table.** Amino acid mutations associated to 13741 sequences of the Spike protein available on May 08 in COVID-19 Viral Genome Analysis Pipeline, enabled by data from GISAID.
