# Supplementary material for: Modelling conformational state dynamics and its role on infection for SARS-CoV-2 Spike protein variants
Source: PLoS Comput Biol. 2021 Aug 5;17(8):e1009286. doi: 10.1371/journal.pcbi.1009286 (PMC8384204; doi:10.1371/journal.pcbi.1009286)
Supplement: S2 Table — (DOCX) [file pcbi.1009286.s009.docx]

| Random 1 | L1145F, A1080F, H1088Q |
| --- | --- |
| Random 2 | I402A |
| Random 3 | F906N, G1035V |
| Random 4 | L865E, N234Y |
| Random 5 | G103H, T881L |
| Random 6 | N74D, L242V, S161W, L335M |
| Random 7 | R1019K, Y636F, L611A, G889I |
| Random 8 | D820R, V213N |
| Random 9 | G971H, G683I, V635P |
| Random 10 | L223M, Q690V, V736C |
| Random 11 | N343C, D290Q, I472P |
| Random 12 | Y741F, S929P |
| Random 13 | F888M |
| Random 14 | N149I, L270S |
| Random 15 | P412H |
| Random 16 | Y365I |
| Random 17 | N17W |
| Random 18 | V1060F, P600L |
| Random 19 | P57Q, V915W, L84W |
| Random 20 | F797A, Q1010W, D1118N |
| Random 21 | T167E |
| Random 22 | Q1005L, A771L |
| Random 23 | T240Y, V656I, F592T, L828K |
| Random 24 | K113Q, Q506H, M697F |
| Random 25 | T599E, E281V, W1102M, N331Y |
| Random 26 | L118C, P330K, F55P |
| Random 27 | I850L, S673R, F1052H, L216P |
| Random 28 | Q414S, P1140Q |
| Random 29 | D737S, W353E, F175R |
| Random 30 | R328G, V512G, E96M, K557H |

**S2 Table.** Random mutants accumulating from one to four mutations.
