# Supplementary material for: Modelling conformational state dynamics and its role on infection for SARS-CoV-2 Spike protein variants
Source: PLoS Comput Biol. 2021 Aug 5;17(8):e1009286. doi: 10.1371/journal.pcbi.1009286 (PMC8384204; doi:10.1371/journal.pcbi.1009286)
Supplement: S3 Table — (DOCX) [file pcbi.1009286.s010.docx]

| Variant | FDS | Predicted Occupancy | | |
| --- | --- | --- | --- | --- |
|  |  | Open state | Closed State | Difference (Open – Closed) |
| Q14H | 0.345 | 25.836% | 74.164% | -48.328% |
| N61P | 0.324 | 25.835% | 74.165% | -48.330% |
| K417I | 0.467 | 25.806% | 74.194% | -48.389% |
| P491H | 0.374 | 25.804% | 74.196% | -48.391% |
| R355Y | -0.632 | 25.772% | 74.228% | -48.456% |
| Q14F | 0.350 | 25.765% | 74.235% | -48.470% |
| Y369N | 0.379 | 25.731% | 74.269% | -48.538% |
| K417C | 0.500 | 25.725% | 74.275% | -48.549% |
| Q409A | 0.403 | 25.719% | 74.281% | -48.562% |
| F486M | 0.310 | 25.658% | 74.342% | -48.684% |
| R355W | -0.541 | 25.648% | 74.352% | -48.705% |
| I231P | -0.566 | 25.622% | 74.378% | -48.756% |
| G416S | 0.331 | 25.503% | 74.497% | -48.994% |
| K417M | 0.310 | 25.467% | 74.533% | -49.066% |
| G416N | 0.306 | 25.319% | 74.681% | -49.362% |
| P230Y | -0.547 | 25.248% | 74.752% | -49.505% |
| Y489M | 0.422 | 25.168% | 74.832% | -49.663% |
| D111W | -0.547 | 25.162% | 74.838% | -49.677% |
| F464L | -0.575 | 25.030% | 74.970% | -49.941% |
| E465W | 0.361 | 24.977% | 75.023% | -50.046% |
| E465K | 0.322 | 24.896% | 75.104% | -50.208% |
| E465Y | 0.391 | 24.798% | 75.202% | -50.405% |
| D111F | -0.656 | 24.782% | 75.218% | -50.436% |
| Y369V | 0.382 | 24.711% | 75.289% | -50.578% |
| L368N | 0.339 | 24.650% | 75.350% | -50.700% |
| L368G | 0.330 | 24.581% | 75.419% | -50.838% |
| P230D | -0.683 | 24.515% | 75.485% | -50.969% |
| L368S | 0.337 | 24.336% | 75.664% | -51.329% |
| L368C | 0.358 | 24.173% | 75.827% | -51.654% |
| S469W | -0.523 | 23.995% | 76.005% | -52.011% |
| L368A | 0.383 | 23.954% | 76.046% | -52.091% |
| L368P | 0.331 | 23.829% | 76.171% | -52.342% |
| Y369E | 0.501 | 23.744% | 76.256% | -52.513% |
| E465A | 0.360 | 23.694% | 76.306% | -52.612% |
| E465S | 0.336 | 23.051% | 76.949% | -53.897% |
| G404Y | 0.414 | 22.941% | 77.059% | -54.117% |
| V503D | 0.303 | 22.614% | 77.386% | -54.773% |
| G404H | 0.341 | 22.321% | 77.679% | -55.359% |
| R403N | 0.331 | 21.822% | 78.178% | -56.356% |
| R403P | 0.308 | 21.716% | 78.284% | -56.568% |
| N394K | -0.524 | 21.586% | 78.414% | -56.828% |
| G404N | 0.411 | 21.544% | 78.456% | -56.911% |
| Y421G | 0.455 | 14.552% | 85.448% | -70.896% |
| G232E | -0.707 | 11.578% | 88.422% | -76.844% |
| G232S | -0.686 | 8.996% | 91.004% | -82.008% |
| G232V | -0.713 | 5.508% | 94.492% | -88.983% |
| G232P | -0.647 | 5.042% | 94.958% | -89.917% |
| G232C | -0.664 | 3.065% | 96.935% | -93.870% |
| G232M | -0.609 | 3.033% | 96.967% | -93.935% |
| G232Q | -0.688 | 2.773% | 97.227% | -94.454% |
| G232T | -0.602 | 1.350% | 98.650% | -97.300% |

**S3 Table.** Putative mutations and associated VDS (ΔS_vib (open)_ – ΔS_vib (close)_, in units of J.K^-1^) and predicted occupancies with open occupancies lower than for the wild type for the 64 mutants with VDS>0.3 and the 20 mutants with lowest VDS values.
