# Supplementary material for: Modelling conformational state dynamics and its role on infection for SARS-CoV-2 Spike protein variants
Source: PLoS Comput Biol. 2021 Aug 5;17(8):e1009286. doi: 10.1371/journal.pcbi.1009286 (PMC8384204; doi:10.1371/journal.pcbi.1009286)
Supplement: Attachment — Submitted filename: Teruel_SARS-CoV-2_Spike20210708_Response.pdf [file pcbi.1009286.s011.pdf]

Université de Montréal

Faculté de médecine
Département de pharmacologie et physiologie

July 9, 2021

To: Dr. Roland Dunbrack Jr. & Dr. Arne Elofson,
Editors Plos Computational Biology

From: Dr. Rafael Najmanovich
Dept. Pharmacology & Physiology
University of Montréal

Subject: Response to reviewers manuscript PCOMPBIOL-D-21-00189

Dear Roland, Arne,

Thank you for the reviews for our manuscript titled "Modelling conformational state dynamics and its role on infection for SARS-CoV-2 Spike protein variants" after an unresonably lenghty review from January to mid May. In light of the speed that SARS-CoV-2 research has evolved during the pandemic the most serious concerns by the reviewers have in fact been addressed by others while the manuscript was being reviewed and serve as validation of our methodology. Specifically, the request to perform Molecular Dynamics (MD) simulations to validate the hypothesis that the D614G mutation does indeed favour the open conformation as well as our choice of ignoring the glycans in Spike. As it turns out, MD simulations do in fact validate our hypothesis and as the simulations were performed with glycans and show that the open state is favoured by the mutation, this result implicitly validates our simplification. As far as explicitly simulating glycans within our coarse-grained models, while it may be possible to do so, it is a study on its own right that is well beyond the scope of the present study and unlikely to change any conclusions given the MD results (and experimental results discussed next). During the time since this work appeared as a preprint and was submitted for review, also **experimental work has appeared confirming our results of higher occupancy of the open state**. Not only for the D614G mutant (in the two publications highlighted by reviewer 2) but also for the B.1.1.7 (UK), 501.V2 (SA) and P1 (Brazil) variants (Gobeil *et al., 2021*). All these are papers are discussed in the revised manuscript as described below.

Whereas experimental and computational work validating our conclusions has appeared while this manuscript is under review, its acceptance and promt publication are still timely and of utmost importance. This is so because this work provides a theoretical and computational framework to understand the mechanism underlying the effect of these mutations and as the pandemic is still far from over, new variants may still appear and our high-throughput results can contribute to signal future emerging variants as variants of concern. Furthermore, from a computational biophysics standpoint the is seminal in introducing the application of normal mode analysis to understand conformational transtions and the effect of mutations thereof in a high-throughput manner.

We ask you to consider the changes we implemented as described below and to accept the manuscript as fast as possible given to ongoing pandemic with new strains continuing to appear (such as Delta+). We believe that the manuscript does not requires an additional round of reviews given the importance of the results and the satisfactory level of validation that our results have independently obtained. On a personal note (Rafael Najmanovich), not often in my career have I seen computational work, particularly with coarse-grained simplified models, that agrees to this extent with experimental results. I think this is a paper that, just like the preprint that is on the top 5% of interest among all tracked literature with 22 citations in less than a year, will be a paper that Plos Computational Biology will be proud to count among its publications for years to come. Please see below the detailed answers to the reviewers comments (in blue/italic).

C.P. 6128, succursale Centre-ville
Montréal QC H3C 3J7
Téléphone : +1 (514) 343-6345
Télécopieur : +1 (514) 343-2291
rafael.najmanovich@umontreal.ca
www.umontreal.ca

Université de Montréal

Faculté de médecine
Département de pharmacologie et physiologie

Reviewer #1: The manuscript describes an interesting and timely computational study of SARS-CoV-2 Spike protein variants and their impact on infectivity. The results will be very useful not only for the scientific community but also for the public health systems. The methodology presented could be of great help to predict the risk of new strains.

It's well written. It would however beneficiate from a thorough review:

Thank you very much for the comment. As the reviewer states that it is a well written manuscript but would benefit from a thorough review, we did just that. We improved the clarity considerably in the revision.

- Page 9: Explain the meaning of "May 08"

This first evaluated variants, used for the clustering of Dynamical Signatures in Figure 3, were based on the sequences available at GISAID on this date. Currently, with the spreading of the pandemic and the increasing genome sequencing of the new coronavirus, we are reaching 2 million sequences available at GISAID. At the time of those first results, we worked only with the 13741 sequences available. The meaning of date is now more clearly explained in the manuscript.

- In Figure 3, it is not clear which data are represented in the y-axis.

The Y-axis represents the Euclidean distance between the Dynamical Signatures of the mutants evaluated as part of that analysis. This point was clarified within the text in page 9 and in the legend of Figure 3. The figure itself was also redone in higher resolition and the branches of interest were highlighted.

- Figure 8: Results showed in figure 8 should be better explained in the main text because there are some inconsistencies. Figure legend should be revised.

Figure 8 shows examples of Dynamical Signatures for single mutants among the top (K417E, G252M and G404Y) and bottom (R355Y, F464L and I231P) $\Delta\Delta S_{vib}$ scores (now for clarity defined as VDS or Vibrational Difference Score) to illustrate that VDS represents changes in flexibility in several areas of the protein in the closed state, the open state, or both. This figure is not fundamental for the narrative of the article, so it was moved to Supplementary Materials to improve the clarity and narrative flow of the article. The axis and the legend were revised.

- It is not clear the usefulness of 3.5 section

Section 3.5 was not well connected to the rest of our narrative and therefore was removed. Nevertheless, the observations on the influence of immune escape on the emergence of new variants are important for the evaluation of mutants and were added to section 3.4. Section 3.4 was greatly revised and new data was added for additional variants of concern that appeared more recently, namely the Brazil strain P1, as well as the Indian Delta (B.1.617.2) and Delta+ (AY.1) strains.

- 3.6 section should be better included in Material & Method section

C.P. 6128, succursale Centre-ville
Montréal QC H3C 3J7
Téléphone : +1 (514) 343-6345
Télécopieur : +1 (514) 343-2291
rafael.najmanovich@umontreal.ca
www.umontreal.ca

Université de Montréal

Faculté de médecine
Département de pharmacologie et physiologie

A new section with instructions to use the data visualization tools have been added as section 2.3 within Materials & Methods.

- A final conclusion should be included. Actual conclusions are more like a summary of the manuscript.

We added a Conclusion – section 4 – summarizing the manuscript and also addressing some new findings that provide a higher level of trust to our conclusions (see below as part of Reviewer #2 concerns).

Reviewer #2: In the manuscript "Modelling conformational state dynamics and its role on infection for SARS-CoV-2 Spike protein variants" the authors utilize coarse-grained normal mode analyses to model the dynamics of Spike proteins and calculate transition probabilities between states for a number of Spike variants. The results predict an increase in open-state occupancy for the more infectious D614G via an increase in flexibility of the closed-state and decrease of flexibility of the open-state. The manuscript also presents a high throughput analysis of simulated single amino acid mutations on dynamic properties to seek potential hotspots and individual Spike variants that may be more infectious. The authors introduce a Markov model of occupancy of molecular states with transition probabilities derived from our analysis of dynamics that recapitulates experimental data on conformational state occupancies.

The biological problems addressed in this work are of clear fundamental and therapeutic interest and insights from computational approaches are certainly welcome to improve our understanding of the SARS-CoV-2 spike mechanisms and interactions.

We would like to thank the reviewer for the thorough analysis of our work. As can be seen from our responses below all concerns were addressed.

Major points:

1.This is a fairly well-executed technical study describing an interesting combination of computational simulation tools to understand mechanisms of SARS-CoV-2 spike proteins in the native and mutant states. Although some of the presented results are certainly very interesting, the manuscript lacks organization, structure and a clearly formulated methodological objective. The overall presentation of the results is fragmented making difficult to understand the logic and methodological details of this work.

We highly appreciate the comment that this is a fairly well-executed study. As discussed above we improved the organisation, structure and clarity.

2. There is an enormous literature about this manuscript (both computational and experimental) that is only very briefly mentioned in Introduction. The authors should have critically assessed the previous studies and, more importantly, identify key issues and questions unanswered thus far.

We have critically assessed previous studies within the extremely long timeframe of the revision that this manuscript underwent. We added additional references to both experimental and computational studies that are relevant. The underlying hypothesis of the variable changes in flexibility to the open and closed states as leading to a higher occupancy of the open state and that in turn correlating with the spread of variants of concern is still holding and is relevant in that it offers a molecular mechanisms that contributes in part to the success of variants of concern.

3) The performed CG simulations do not apparently include the glycosylation of the spike, therefore strongly reducing the biological relevance of the entire work. Perhaps the authors should consider a model to mimic the glycosylated microenvironment in the framework of CG approaches. Although glycans are not supported by many CG methods

C.P. 6128, succursale Centre-ville
Montréal QC H3C 3J7
Téléphone : +1 (514) 343-6345
Télécopieur : +1 (514) 343-2291
rafael.najmanovich@umontreal.ca
www.umontreal.ca

which represents an important limitation in adopting this specific computational approach to study fusion viral proteins where glycosylation plays a key role, there exist CG methods (such as Martini) where the energetic parameters have been recently extended to N-glycans. Glycans have been widely found to be crucial in the modulation of the spike conformational dynamics and should be considered for modeling of spike proteins. The inclusion of glycans to the model could potentially change the results and this comparison would be very important to substantiate the main findings and conclusions.

The reviewer rightly notes that we make an important simplification in our work. Namely, that we ignore the glycans that are part of the Spike glycoprotein. We do clearly state in the manuscript this simplification but it must be emphasized that despite this simplification, our predictions were verified by experimental and computational Molecular Dynamics simulations performed while the manuscript has been in review. Our work is entirely based on a normal mode analysis. The proposed Coarse-Grained analysis (Martini) is based on Molecular Dynamics and Martini specifically is a force field, NOT a method in itself and less so a normal mode analysis method. The Martini force field can be used in the context of coarse-grained molecular dynamics simulations, which are out of the scope of the methods used in this paper as coarse grained molecular dynamic simulations would still be too slow to perform the 17081 single mutant simulations we performed with our coarse-grained normal mode analysis method. Returning to the simplification of ignoring glycans, we believe that our level of coarse-graining makes the effects of the glycans negligible. Furthermore, the most reliable full atom long molecular dynamics simulations performed in the presence of glycans validate our results (Mansbach *et al.,* 2021) as do experimental results (Zhang *et al.,* 2021; Benton *et al.,* 2021; Gobeil *et al.,* 2021). Two paragraphs were added to the new conclusions section discussing this.

4) The authors claim that their results correctly model an increase in open-state occupancy for the more infectious D614G via an increase in flexibility of the closed-state and decrease of flexibility of the open-state.

There have been recently cryo-EM structures of a full-length G614 trimer (Structural impact on SARS-CoV-2 spike protein by D614G substitution. Science 2021, 372, 525-530) for distinct prefusion conformations in the closed, intermediate and 1-up open states (pdb ids 7krq, 7krs, 7krr) that characterized previously disordered regions in spike protein. This study supported the reduced shedding mechanism and suggested the increased stability of the G614 mutant. At the same time, another recent work in PNAS (The effect of the D614G substitution on the structure of the spike glycoprotein of SARS-CoV-2. Proc. Natl. Acad. Sci. U. S. A. 2021, 118, e2022586118, pdb ids 7bnm, 7bnn, 7bno). These G614 mutant structures were more flexible and wide-open which is in line with the increased flexibility of the open state as proposed in the reviewed manuscript. These studies proposed different mechanisms, but it may reflect the diversity of conformational states adopted by the G614 mutant spike trimer.

Given simplicity of the elastic network models, I would suggest testing these structures (or at least some of them) to try to reconcile conflicting mechanisms and also understand the effect of the G614 structures on the results and predictions.

Thank you very much for the suggestion. Even though the Science article speculates about the relation between infectivity and trimer stability, with respect to the results regarding occupancy **both, the Science and PNAS papers mentioned agree with the conclusions in our manuscript**, namely that the open state has higer occupancy. Specifically: "… *This observation can explain why the G614 trimer favors the DBD-up conformations*." (Zhang *et al.*, Science 2021); And "*Strickingly, the structures we solved (Table 1) show that the G614 spike exists predominantly in the open, receptor-binding-competent conformation, in*

contrast to the D614 spike…" (Benton *et al.*, PNAS 2021). Both papers came to out attention as this manuscript was still under review and their structures were analysed. It is important to note that both pairs of structures have a lower resolution than those we used in our study. In addition to that, we still have to model missing loops and residues to be able to use the structures. We utilized the open/closed pairs 7KRR/7KRS (Zhang *et al.*, Science 2021) as well as 7BNN/7BNM (Benton *et al.,* PNAS 2021) to obtain open state occupancies of 82% and 63%, respectively. Zhang *et al.* (7KRR/7KRS) do not provide specific occupancy values. However Benton *et al.,* calculate the open state occupancy as 83% (63% in our predictions with their structure) for G614 and 17% for D614 (25.8% in our predictions). It is unclear to what extent the modelling errors and lower resolution of the structures of the D614G mutant impact the results. However, as the core of the work involves modelling mutations throughout the structure, our approach of using the same reference structures (6VYB and 6VXX) for all modelled mutations is correct in our view. We discuss the agreement between the conclusions of the two papers above in the Conclusions section.

Additionally, a third paper appeared recently (Gobeil *et al.,* Science 2021) determining the structure of the B.1.1.7 (UK), 501.V2 (SA) and P1 (Brazil) strains and determining the occupancy preferences between states. In all cases showing an increase of propensity for the open state – also in agreement with our computational results based on modelling the structures of these variants.

5. Could the authors more clearly identify what makes their findings novel to biological community? What do the results of this study add to our current knowledge of the role of protein dynamics in these mechanisms?

This work adds to our current knowledge in a number of ways. First, it provides a computational framework to simulate (and therefore explain) the experimental results of occupancy. Second, the high-throughput predictions are still valid as the pandemic is still going on and the virus continues to evolve. It is important to clarify that evaluations of dynamic properties have never been previously done at a high-throughput level (over 17,000 mutants). Third and more generally, from a computational biophysics standpoint, this work demonstrates that it is possible to estimate the effect of mutations in conformational state occupancies with a simplified model amenable to high-throughput analysis. This is something that can have ample applications in diverse fields such as signalling and allosteric modulation.

6. It would be desirable to also use all-atom MD simulations for some of the studied systems to allow for a comparative analysis of protein flexibility. In general, the analysis of computational simulations are not sufficiently justified which weakens their connection with the biological evidence.

We have included a paragraph about this in the conclusion. All-atom MD simulations are indeed a desirable way to study protein flexibility. However, in the case of a big system like the Spike protein, they need to be conducted for sufficient timescales in order to be of any use. For example, the work we cite in the conclusion (Benton *et al., 2021*) has cumulated 20 µs of simulation time, which on a big system like Spike probably represents several hundred

C.P. 6128, succursale Centre-ville
Montréal QC H3C 3J7
Téléphone : +1 (514) 343-6345
Télécopieur : +1 (514) 343-2291
rafael.najmanovich@umontreal.ca
www.umontreal.ca

CPU-years, all for the comparison of two sequences: WT and D614G. This is the longest and most scientifically sound MD simulation for the Spike protein WT and D614G mutants. Our approach, for all the 17081 mutations studied, necessitated 0.65 CPU-year. Since the work cited above confirms our results, we are confident that our model captures the essential long-timescale dynamics of the Spike protein.

7. I believe that the authors should spend some time thinking how to strengthen the interface between experiment and computations in the manuscript to substantiate key findings.

We did a new bibliographical review, including new and recent publications, and it showed the key importance of our work when comparing our results with the new experimental findings. All the novel experimental evidence that appeared after our preprint was submitted and sent for review validates our conclusions. All these references were added to the Conclusions section.

8) Although the system is fascinating and computational approach is generally appropriate, the manuscript often reads as a set of disconnected observations rather than a cohesive story with the detailed analysis and insightful discussion.

We do thank the reviewer to highlighting the issue and we tried to improve the presentation. As mentioned above we improved the clarity of the manuscript by adding new text, modifying existing text and rearranging some of the material presented. I should add that whereas we would wish to be able to satisfy the personal taste on how scientific facts are presented in a manuscript for every possible reader of our manuscript, it is an impossible task.

9) The results often lack proper interpretation and integration with experiment to justify findings.

Wherever new experimental data became available since the original submission of the manuscript we have integrated it to the text.

10). I believe that the authors should spend some time thinking how to strengthen the interface between experiment and computations in the manuscript to substantiate key findings.

As discussed above, new experimental data that appeared while the manuscript was under revision has been added where relevant. It is important to note that all experimental evidence that existed up to November 2020 was taken in consideration such as for example the experimental data on occupancy of variants containing the D614G mutation that appered after our initial hypothesis of the importance of dynamics to the association between Spike and ACE2 in June 2020. We also added in this revision the experimental studies that appeared during the 5 months that this manuscript has been under review.

Minor points:

1. The illustrations are often not sufficiently informative and generally very poor. Many of the plots and data cannot

C.P. 6128, succursale Centre-ville
Montréal QC H3C 3J7

Téléphone : +1 (514) 343-6345
Télécopieur : +1 (514) 343-2291

rafael.najmanovich@umontreal.ca
www.umontreal.ca

Université de Montréal

Faculté de médecine
Département de pharmacologie et physiologie

be seen at all. The authors should redesign and redo most of these figures and make them better organize, visible and informative with necessary annotations.

The figures that the reviewer might have had access to were probably of lower resolution for some reason unknown to us. We will make sure that the figures submitted for publication are of sufficient quality. Some of the plots were redesigned.

2. The manuscript is lacking a systematic statistical framework for assessing significance and quality of predictions. The authors should more clearly formulate and apply their statistical instruments along a common strategy to provide more confidence of quality and reproducibility of their results.

The determination of parameters for the calculation of occupancies are associated with highly significative p-values. Out of the over 17000 mutants, we discuss those with the highest $\Delta\Delta S_{vib}$ (VDS) values, therefore associated to highest z-scores. The presentation of actual z-scores would be of no consequence as the threshold of $\Delta\Delta S_{vib}$ (VDS) was arbitrarily defined by our capacity to perform occupancy calculations.

Kind regards,

Rafael Najmanovich

Rafael Najmanovich, PhD.
Full Professor
Department of Pharmacology and Physiology
